# Effects of early, combined endurance and resistance training in mechanically ventilated, critically ill patients: a study protocol for a randomised controlled trial

**DOI:** 10.1186/s13063-016-1533-8

**Published:** 2016-08-15

**Authors:** Sabrina Eggmann, Martin L. Verra, Gere Luder, Jukka Takala, Stephan M. Jakob

**Affiliations:** 1 Department of Physiotherapy, Inselspital, Bern University Hospital, Bern, 3010 Switzerland; 2 Department of Intensive Care Medicine, Inselspital, Bern University Hospital and University of Bern, Bern, 3010 Switzerland

**Keywords:** Physiotherapy, Physical function, Intensive care, Mechanical ventilation, Rehabilitation, Weakness, Critical illness

## Abstract

**Background:**

Prolonged need for intensive care is associated with neuromuscular weakness, termed Intensive Care Unit Acquired Weakness. Those affected suffer from severe functional impairment that can persist for years. First studies suggest a positive effect of physiotherapy and early mobilisation. However, the ideal intervention for a preferential functional outcome is not known. So far no randomised controlled trial has been conducted to specifically evaluate an early endurance and resistance training in the mechanically ventilated, critically ill patient.

**Methods/design:**

A randomised controlled trial with blinded assessors and 6-month follow-up will be conducted in a tertiary, interdisciplinary intensive care unit in Switzerland. Participants (*n* = 115; expected dropouts: *n* = 15) will be randomised to a control group receiving standard physiotherapy and to an experimental group that undergoes early mobilisation combined with endurance and resistance training. The inclusion criteria are being aged 18 years or older, expected mechanical ventilation for more than 72 h and qualitative independence before the illness.

Primary endpoints are functional capacity (6-Minute Walk Test) and the ability to perform activities of daily living (Functional Independence Measure) measured at hospital discharge. Secondary endpoints include muscle strength (Medical Research Council sum score, handgrip strength and handheld dynamometry for quadriceps muscle), joint contractures (range of motion), exercise capacity (Timed ‘Up & Go’ Test) and health-related quality of life (Short Form 36). Safety will be monitored during interventions by indirect calorimetry and continuous intensive care standard monitoring. All previously defined adverse events will be noted. The statistical analysis will be by intention-to-treat with the level of significance set at *p* < 0.05.

**Discussion:**

This prospective, single-centre, allocation-concealed and assessor-blinded randomised controlled trial will evaluate participant’s function after an early endurance and resistance training compared to standard care. Limitations of this study are the heterogeneity of the critically ill and the discontinuity of the protocol after relocation to the ward. The strengths lie in the pragmatic design and the clinical significance of the chosen outcome measures.

**Trial registration:**

German Clinical Trials Register (DRKS): DRKS00004347, registered on 10 September 2012.

## Background

Intensive care unit (ICU) survivors have a persistently high mortality and an exceedingly poor quality of life that manifests itself up to 5 years after ICU discharge [1]. Further burdens include exercise limitation, physical and psychological complications as well as increased costs and use of health care services [2]. To outline these multiple long-term consequences after survival of a critical illness the term post intensive care syndrome (PICS) has been agreed on [3]. Consequently, PICS describes impairments in physical, cognitive or mental health status subsequent to a critical illness and persisting beyond hospital discharge.

### Intensive Care Unit Acquired Weakness (ICUAW)

Neuromuscular dysfunctions are an important aspect of the physical impairment following ICU stay and are probably a crucial contributor to the associated long-term disability. They are common in critically ill patients requiring more than 1 week of mechanical ventilation and have an approximate incidence of 40 % [4]. A simple framework to diagnose and classify these neuromuscular disorders has been proposed [5]. Hence, the term ‘Intensive Care Unit Acquired Weakness’ (ICUAW) describes a clinically detected weakness in critically ill patients with no plausible aetiology except for the critical illness itself. ICUAW is associated with worse outcomes such as prolonged weaning from mechanical ventilation [6], weaning failure with a high occurrence of reintubation or tracheotomy [7], longer ICU and hospital stays [8], increased ICU and hospital mortality [9], increased mortality 180 days after ICU discharge [10] in addition to poor functional status, persistent disability in activities of daily living, reduced walking ability and lower quality of life up to 1 year after ICU discharge [11–14] The underlying pathophysiological mechanisms of ICUAW are complex and poorly understood. Several risk factors, in particular, high severity of illness with multiorgan failure, hyperglycaemia and immobilisation, contribute to the development of ICUAW [15]. The process of muscle wasting begins during the first week of a critical illness and thus commences very early and rapidly [16]. So far there is no specific treatment for ICUAW. However, the potential benefit of early rehabilitation and physiotherapy has been suggested by a recent Cochrane review [17].

### Rehabilitation in intensive care

Physiotherapists are seen as an essential part of the multidisciplinary ICU team [18]. Compared to other health care professionals they can be more successful in facilitating early mobilisation and rehabilitation in critically ill patients [19]. Their specific knowledge of neurological and musculoskeletal conditions enables the physiotherapist to assess current rehabilitation needs, to detect early deficits, such as ICUAW, and to set rehabilitation goals accordingly [18]. Consequently, physiotherapists seem ideal to meet the recommendations of the National Institute for Health and Care Excellence (NICE) for an individualised, structured rehabilitation programme with frequent follow-up reviews during critical illness [20]. However, there are only a few randomised controlled trials to support this statement. One randomised controlled trial was conducted by Burtin and colleagues in a medical and surgical ICU and involved 90 patients with a prolonged ICU stay [21]. Participants were randomised into standard physiotherapy alone and standard physiotherapy plus an additional bed cycling training 5 days a week. At hospital discharge the 6-minute walking distance, isometric quadriceps force and the Physical Functioning score of the Short Form 36 (SF-36) were significantly higher in the cycling group. Similarly, Schweickert and colleagues conducted a randomised controlled trial in a medical ICU with 104 mechanically ventilated patients [22]. They found that physiotherapy, specifically very early exercise and mobilisation, during periods without sedation led to more patients returning to functional independence at hospital discharge compared to usual care, and led to fewer days with delirium and mechanical ventilation. However, another randomised controlled trial with 150 participants comparing usual care to intensive exercise that started 5 days after ICU admission, continued during the stay in the ward and for 8 weeks in an outpatient setting, failed to show any benefit for intensive exercise therapy [23]. Finally, a randomised controlled trial, including 50 patients with sepsis syndrome, compared an individualised physical rehabilitation programme starting within 48 h after sepsis diagnosis to standard care without active rehabilitation [24]. There was no significant difference in physical function at ICU discharge, but individuals in the intervention group reported an improvement in physical function and physical role 6 months after hospital discharge on the SF-36.

Given the discrepancies of the findings in the previous few studies, further research to evaluate the long-term outcome of early rehabilitation interventions seems necessary. Future studies should be performed with high methodological quality and report the administered frequency, intensity and timing of the interventions [17, 25]. To our knowledge no randomised controlled trial has so far been conducted to evaluate the efficacy of early physiotherapeutic interventions, including both an endurance and resistance component, in the mechanically ventilated, critically ill patient. Aerobic and strength training are widely recommended and are effective interventions in chronic diseases and disabilities like stroke [26], chronic heart failure [27], older adults’ disabilities [28], chronic obstructive pulmonary disease [29] and cancer-related fatigue [30]. We expect a similar benefit in critical illness. To avoid the limitations of previous studies, we aim to include participants from a general ICU population, containing patients at risk of a prolonged ICU stay and ICUAW as a consequence. Also, and because ICUAW develops early and rapidly, the goal is to start early with low-intensity exercise that will be increased according to the patient’s level of tolerance until full mobilisation.

### Study purpose

This study aims to investigate the effects and safety of an early endurance and resistance training combined with early mobilisation in comparison to standard care in critically ill, mechanically ventilated adults at an interdisciplinary, tertiary ICU.

### Hypotheses

The primary hypothesis is that critically ill, mechanically ventilated adults who participate in an early endurance and resistance training, combined with early mobilisation, have an improved functional capacity and are, therefore, more functionally independent at hospital discharge compared to patients receiving the usual physiotherapy care. Secondary hypotheses are that this early training is as feasible and as safe as standard therapy. Further, we expect improvements in muscle strength at ICU discharge, fewer joint contractures, less time on mechanical ventilation, a shorter length of stay in the ICU and the hospital, and a higher quality of life at 6 months after hospital discharge compared to patients receiving usual care.

## Methods and design

**Fig. 1:**
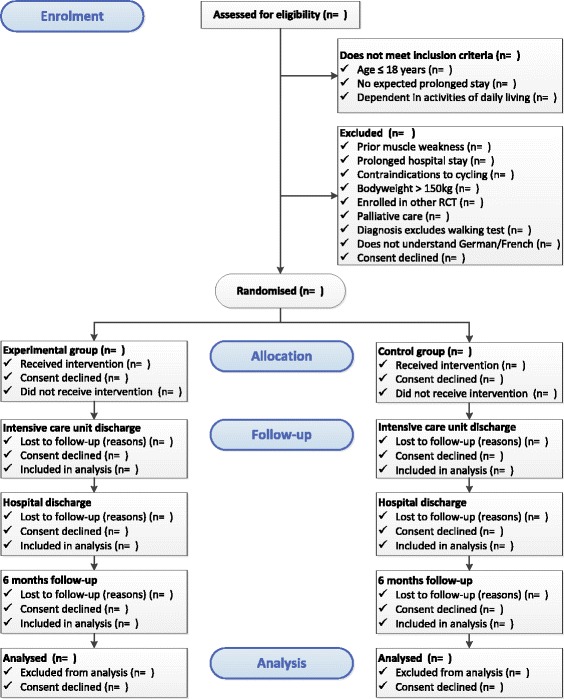
Study flow diagram (CONSORT) [31]. Legend: RCT, randomised controlled trial

We will conduct a prospective, single-centre, allocation-concealed and assessor-blinded randomised controlled trial with superiority design and 6-month follow-up. The study has been approved by the Ethics Committee of Canton Bern (KEK), Switzerland and is registered in the German Clinical Trials Register (DRKS00004347). This trial adheres to the recommendations from the Consolidated Standards of Reporting Trials (CONSORT) statement [31] (Fig. 1) and is conducted according to Swiss law and Good Clinical Practice (GCP) guidelines.

### Study population

The study is being conducted in the tertiary, interdisciplinary ICU of the Department of Intensive Care Medicine at the Inselspital, Bern University Hospital. To be eligible to participate in the study patients must be aged 18 or older, be expected to stay on mechanical ventilation for at least 72 h, which reflects a prolonged stay at our unit, and finally, participants must have been independent in their activities of daily living before the onset of critical illness (verbal statement by their proxy). Patients with prior muscle weakness, such as a preexisting neurological or neuromuscular disease with functional deficits or a prolonged stay in the hospital for at least 10 days prior to the ICU admission, are excluded from the study. Further exclusion criteria are contraindications to cycling (mainly fractures or recent surgical procedures to the lower limbs, acute thrombosis, preexisting open wounds, extracorporeal membrane oxygenation (ECMO) and body weight of more than 150 kg), patients who are already enrolled in another intervention study, patients receiving palliative care, patients with a diagnosis on admission that excludes the possibility of walking at hospital discharge (for example, paraplegia) and lastly patients who do not understand either German or French.

### Standard care (control group)

Participants randomised to the control group will receive usual physiotherapy and ICU standard care, which includes sedation and weaning protocols based on previous publications [32]. Current physiotherapy practice is comparable to the European norm [33] consisting of positioning, respiratory therapy, passive range of movement exercises for nonresponsive patients or active exercises if arousable, and early mobilisation. In order to start as early as possible, physiotherapists screen patients regularly focussing on prevention and treatment of functional and pulmonary impairment. However, subject to our internal procedure, physiotherapy and mobilisation will start after medical prescription. Treatments are based on the therapist’s assessment and are accordingly individually tailored. Sessions will usually take place once daily from Monday to Friday and, if deemed necessary (e.g. severe weakness, intensive rehabilitation and weaning period, retained airway secretion or atelectasis in extubated patients), this includes treatment at the weekend.

### Study intervention (experimental group)

**Fig. 2:**
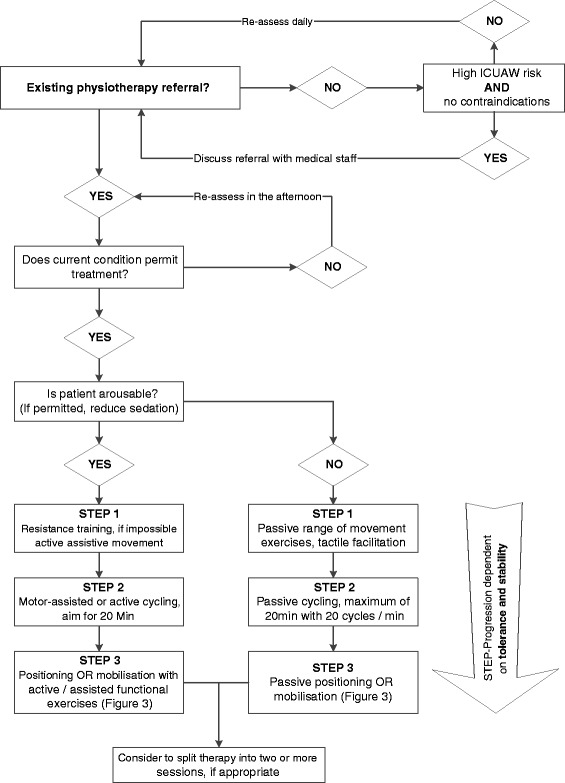
Daily physiotherapy screening and step progression (experimental group). Legend: ICUAW, Intensive Care Unit Acquired Weakness

Patients randomised to the experimental group will receive a standardised exercise programme involving early endurance and resistance training that is combined with early mobilisation. The clinician in charge will decide before study inclusion, whether there are any contraindications to include the patient, and during the study, if at any time the treatment should be stopped, e.g. in case of an adverse event (see safety). Therefore, the intervention will be started as soon as it is deemed safe by the treating ICU team and will occur from Monday to Friday and, if deemed necessary, at the weekend. Throughout all activities, progression will be increased successively, depending on an individual’s tolerance and stability. If needed, the physiotherapist will split the delivery of the exercise programme into one or more sessions (Fig. 2). The number of treatments, its content and duration will be noted for both the control and experimental group.

**Fig. 3:**
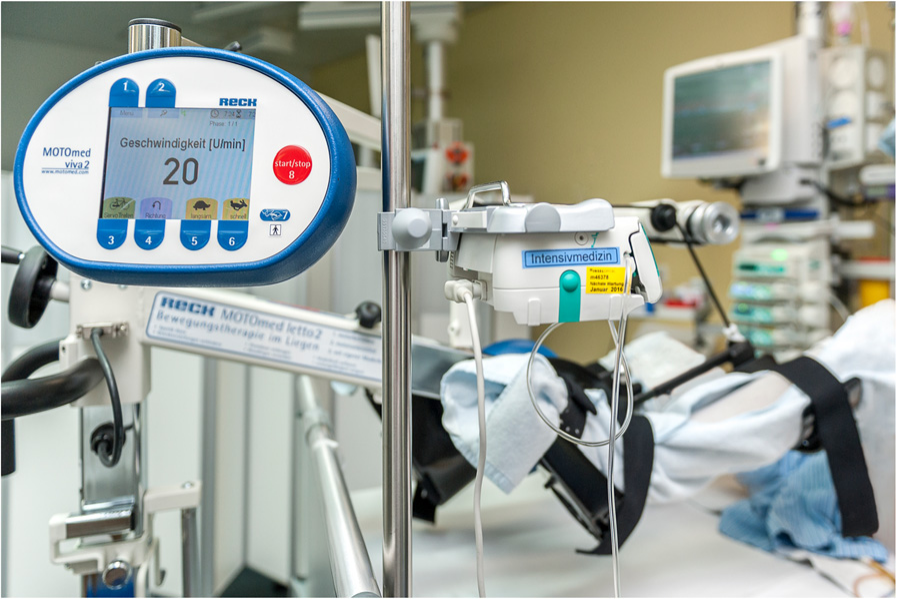
Early endurance training with a bedside cycle ergometer. Picture: Pascal Gugler, Inselspital, Bern University Hospital

Endurance training will be conducted with a motor-assisted bedside cycle ergometer that allows patients to train in a passive, motor-assisted or active mode from a hospital bed (MOTOmed letto2, Reck-Technik, GmbJ & Co. KG, Betzenweiler, Germany) (Fig. 3). Training intensity has been adapted from a previous study with long-term ICU patients from Burtin and colleagues [21]. Thus, the aim for unresponsive patients will be one passive training of 20 min with a fixed pedalling rate of 20 cycles per minute from Monday to Friday. In the course of cycling, patient’s participation will be prompted verbally and by the integrated MOTOmed ServoCycling feature. After achieving patient’s active partaking, the goal will be to train with motor assistance for at least 20 min on level 0. If this is accomplished, assistance will be gradually decreased and subsequently, resistance level and training period increased until a maximum of 60 min on level 6. Tolerance and stability will be judged by the responsible physiotherapist according to patient’s perceived level of exertion (BORG RPE Scale) [34] and especially in unresponsive patients, exceedence of individually set limits. The training position will be supine with an individually adapted head-of-bed elevation to allow for leg movement and comfort.

The resistance training will consist of an individually tailored exercise programme as well as standardised exercises for both upper and lower limbs and will train the muscle groups for shoulder flexion, shoulder external rotation, elbow flexion, hip abduction, knee extension and foot dorsiflexion. The resistance will be administered by the therapist or with weights, starting from 450 g. Training intensity will aim to achieve 50 to 70 % of the estimated one-repetition-maximum, with a goal of 8 to 12 repetitions and 2 to 5 sets with at least a 2-min rest between series. If a participant is able to perform the exercises correctly, he will be given a tutorial with pictures in order to train independently with nurses or family members. If unable to perform the exercises, the physiotherapist will use tactile facilitation or passive range of movement for all joints and their possible directions.

**Fig. 4:**
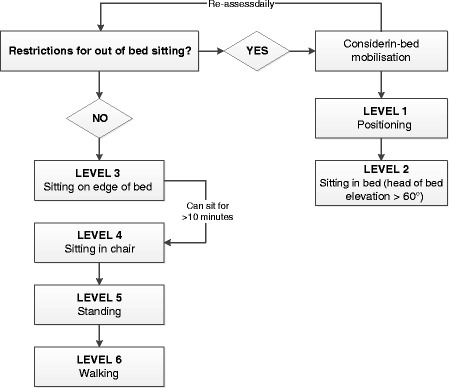
Mobilisation levels (experimental group)

If the previous exercises are well-tolerated, bed mobility and sitting upright in bed will be started (Fig. 4). If these are well-tolerated and no contraindications as per medical prescription exist, the treatment will be further advanced to mobilising participants to the bedside. There, balance exercises will be performed or, if needed, support will be provided by the physiotherapist or various assistive equipment. After being able to sit for at least 10 min on the side of the bed, regardless of whether support is needed, participants will be moved into a chair with an individually adapted transfer according to the individual’s resources. The aim during all mobilisations will be to involve the patient as actively as possible to promote independence. While sitting, functional tasks and activities of daily living will be performed and gradually increased to standing and walking exercises.

### Safety

To ensure the patient’s safety all vital parameters, particularly heart rate, invasive blood pressure and oxygen saturation, will be continuously monitored and recorded by our Patient Data Management System (PDMS), which is routinely checked by the ICU staff. Furthermore, oxygen consumption, energy expenditure and the respiratory quotient by indirect calorimetry will be obtained 30 min prior to, during and for 15 min after physiotherapy. All adverse events (AE) will be noted. These are defined as any event that occurs during or up to 15 min after physiotherapy and persists despite an intervention or therapy interruption. It includes new haemodynamically relevant arrhythmias or otherwise unstable haemodynamics, oxygenation desaturation under 85 %, a fall or other injury as well as any accidental removals of a tube, catheter or similar devices. We use a qualitative physiological approach instead of strict target numbers, as optimal physiological goals for individual patients vary highly and depend on the underlying clinical condition [32]. If a participant exceeds or fails to exceed his individually set limits, the therapist will discontinue the session for the day and will record the reason. If this happens repeatedly, the principal investigator will withdraw the patient from the study. Serious adverse events (SAE) are defined as death, a life-threatening injury or an extension of the length of hospital stay. All SAEs and AEs will be recorded and reported to the principal investigator, who judges their severity and whether they were study-related. All SAEs will also be reported to the local ethics committee. Finally, all occurring complications related to bed rest, such as atelectasis, pneumonia, decubitus ulcer, thrombosis, contracture, delirium and a confirmed critical illness, polyneuropathy or myopathy, will be judged and recorded daily by the treating physician according to our current standard of care.

### Study duration

The study intervention will take place during the entire ICU stay as well as during potential readmissions to the ICU. Therefore, the study duration will be variable for each patient. After ICU discharge both groups will receive the usual physiotherapy treatment and, if necessary, rehabilitation. Likewise, there will be no difference for participants of both groups in terms of medical attention, treatment and care during the whole study duration. The study will finish with a follow-up 6 months after hospital discharge.

### Outcome

The primary outcome measures are functional capacity using the 6-Minute Walk Test (6MWT) [35] and the ability to perform activities of daily living using the Functional Independence Measure (FIM) [36]; both will be evaluated at hospital discharge.

The secondary outcome measures were chosen to reflect the physical impairment after ICUAW and whether these important clinical outcomes can be improved by early training. They are as follows:

Muscle strength and ICUAW diagnosis at ICU discharge: Medical Research Council sum score [5, 37], handgrip strength using the JAMAR dynamometer (Sammons Preston Rolyan, Bolingbrook, IL, USA) [37, 38] and quadriceps muscle strength using a handheld dynamometer (Microfet 2, Biometrics, Almere, Netherlands) [38]. Muscle strength tests will only be performed if a patient can follow three out of five simple commands in order to assess their ability to cooperate as well as the level of consciousness beforehand [39].

Joint contractures, which are defined as limitations in range of motion, will be recorded after ICU discharge for the following joints: shoulder flexion, elbow flexion and extension, fist closure, hip flexion, knee flexion and extension and foot dorsiflexion [40].

Functional mobility will be measured with the Timed ‘Up & Go’ Test [41] at hospital discharge.

The quality of life 6 months after hospital discharge will be assessed with the Short Form 36 (SF-36, version 1.3) [42].

The overall time on mechanical ventilation, length of stay in the ICU and the hospital, as well as achieved milestones during the ICU stay (sitting on the side of the bed, sitting in a chair, standing or walking) will be reported.

Furthermore, the safety of early training, particularly the physiological response of the participants, any occurring AE, SAE and other complications related to bed rest as well as the feasibility, frequency, duration and content of the therapy, will be collected and analysed.

The schedule for the outcome assessments is shown in Table 1.

**Table 1:** Schedule for enrolment, data collection, interventions and outcome measures

	Enrolment	Allocation	Post allocation			
Timepoint	ICU admission		ICU stay (daily)	ICU discharge	Hospital discharge	6-month follow-up
Enrolment:						
Eligibility screen	x					
Consent by nonparticipating doctor	x					
Allocation		x				
Informed consent by proxy		≤72 h				
Data collection:						
Demographic and diagnostic data		x				
APACHE II score	x					
SOFA score			x			
TISS 28 and 76	x			x		
Laboratory findings	x	x		x		
Medication			x			
Physiotherapy Interventions:						
Frequency, duration and content			x			
Safety and physiological parameters			x			
Mobility milestones			x			
Bed rest complications			x			
Serious/adverse events			x			
Outcome measures:						
Muscle strength: MRC sum score, handgrip and quadriceps strength				x		
Range of motion				x		
Functional Independence Measure				x	x	
Length of stay (ICU and hospital), days on mechanical ventilation					x	
6-Minute Walk Test					x	
Timed ‘Up & Go’ Test					x	
Short Form 36						x

*APACHE* Acute Physiology and Chronic Health Evaluation, *ICU* intensive care unit, *MRC* Medical Research Council, *SOFA* Sequential Organ Failure Assessment score, *TISS* Therapeutic Intervention Scoring System

### Recruitment, randomisation and blinding

Patients will be screened daily after their ICU admission for study eligibility by the physiotherapist and research nurse in charge and will be included after consultation with the responsible clinician. Reasons for nonrecruitment will be noted in a daily screening log. If a patient is considered eligible a nonparticipating doctor will reexamine the inclusion and exclusion criteria in order to safeguard the patients’ interests according to Swiss law on nonresponsive patients. Participants will then be randomised at a 1:1 ratio to either the experimental or control group by the responsible research nurse using sequentially numbered, opaque, sealed envelopes to ensure a concealed allocation [43]. The allocation sequence was generated in advance by a study nurse using unrestrictive computer-generated randomisation. Furthermore, according to the recommendations of Good Clinical Practice (GCP) the willingness to participate will be obtained in the form of a presumed consent from the patients’ proxy within 72 h (Table 1). After regaining the power of judgement, patients will be informed of the study in writing and asked retrospectively by a GCP-trained physician to provide written informed consent. Participants have the right to withdraw from the trial at any given time. If unwilling to participate or withdrawing from the trial, patients will be excluded from the study and asked if the data that has already been collected may be retained. The sample size will be adjusted for dropouts.

Due to the nature of the intervention it is not possible to maintain blinding for the treating physiotherapists, ICU staff or participants. However, the group of physiotherapists who will assess the main outcomes will be distinct from the treating therapists and will, therefore, be blinded to group allocation.

### Sample size

For patients with chronic obstructive lung disease the minimal clinically important difference for the 6MWT has been validated at 54 m [44]. Using this threshold and a mean walking distance of 301 m (SD 81) [45] a sample size calculation based on a statistical power of 80 % and an *α*-level of 0.05 requires a sample size of 36 participants per group, i.e. a total of 72 patients for the study. We expect a high correlation between the two primary outcomes and, therefore, only moderately corrected the size upwards by 14 patients each. We assume a dropout rate of 15 % and adjusted the sample size by 15, resulting in a total number of 115 patients. However, we obtained the approval of the Ethics Committee to recruit more patients, if needed, to compensate for a higher number of dropouts (denied informed consent).

### Data collection and management

Table 1 gives an overview of the data to be collected and the time of acquisition, including routinely gathered data such as demographics (age, gender, weight, body mass index), severity of illness (Acute Physiology and Chronic Health Evaluation (APACHE) II score, Therapeutic Intervention Scoring System (TISS28 und TISS76), Sequential Organ Failure Assessment (SOFA) score, diagnostic data as well as laboratory findings and dose and type of sedation, relaxants, vasopressors, opiates, steroids and insulin.

All study data will be collected anonymously with an individually allocated study number on all case report forms and will be managed using REDCap (Research Electronic Data Capture) electronic data capture tools, which is a secure, web-based application designed to support data capture for research studies [46]. Vital parameters for the safety analysis will be collected by the aforementioned PDMS, which allows for continuous data collection and off-line analysis.

### Statistical analysis

Study data will be analysed as randomised with SPSS (IBM SPSS Statistics Version 21) by both intention-to-treat (main analysis) and per-protocol analysis. However, the intention-to-treat analysis will only include participants with an informed consent, as adjusted in the sample size calculation. The level of significance will be set at *p* < 0.05. Descriptive statistics will be used to describe mean outcomes and distribution. Subsequently, the outcome parameters between the two groups will be compared using Student’s *t* tests for normally distributed data and the Mann-Whitney *U* test for nonnormal distribution. For repetitive data repeated measures analysis of variance (ANOVA) will be deployed and for correlations between variables the Spearman correlation coefficients or similar will be used. If missing data are more than 5 %, multiple imputations will be performed. There will be one planned interim analysis after enrolment of 35 patients for assessment of safety.

## Discussion

This prospective, single-centre, allocation-concealed and assessor-blinded randomised controlled trial with 6-month follow-up will be the first, to our knowledge, to evaluate both an early endurance and resistance training compared to standard care in the mechanically ventilated, critically ill patient from a general ICU population. Considering the devastating consequences of ICUAW, it is imperative to find interventions that improve function and thereby quality of life. The two chosen primary outcomes, 6MWT and FIM, reflect these important physical impairments of ICUAW, and thus have a high clinical relevance for patients surviving prolonged mechanical ventilation. Therefore, the strengths of this trial lie in the pragmatic design of the early initiation and physiotherapy-driven protocol, the prospective inclusion of patients at risk of prolonged mechanical ventilation and its consequences in the form of ICUAW in a wide general ICU population that will be screened for study recruitment within 12 h after ICU admission and will, therefore, be enrolled very early into the trial, as well as the everyday significance of the chosen outcome measure to the participants up to 6 months after hospital discharge. If the experimental intervention should prove superior to standard care, it will be relatively simple to incorporate the intervention protocol into daily routine so that it can be used elsewhere or for guideline development. Limitations of this study are the heterogeneity of the critically ill and the discontinuity of the protocol after relocation to the other wards within the hospital. Also, due to the trial’s nature, it is impossible to blind the treating physiotherapists, ICU staff and the participants to the intervention. However, the outcome assessors will be blinded to group allocation. Finally, the reporting of adverse events during therapy will depend on the treating physiotherapists, which could lead to missing events. However, physiotherapists have been carefully trained and work closely with the ICU nurse at the bedside. Moreover, all safety parameters will be continuously recorded by the PDMS for further safety analysis.

## Trial status

The first patient was randomised on 10 October 2012. Recruitment is presently still ongoing, but should be completed by the end of February 2016. Allowing for the 6-month follow-up after hospital discharge, we anticipate the end of the study in August 2016.

## Abbreviations

6MWT, 6-Minute Walk Test; AE, adverse event; APACHE, Acute Physiology and Chronic Health Evaluation; CONSORT, Consolidated Standards of Reporting Trials; FIM, Functional Independence Measure; GCP, Good Clinical Practice; ICU, intensive care unit; ICUAW, Intensive Care Unit Acquired Weakness; NICE, National Institute for Health and Care Excellence; PDMS, Patient Data Management System; PICS, post intensive care syndrome; RCT, randomised controlled trial; REDCap, Research Electronic Data Capture; SAE, serious adverse event; SF-36, Short Form 36; SOFA, Sequential Organ Failure Assessment score; TISS, Therapeutic Intervention Scoring System
